# Audit of Psychotropic Polypharmacy Amongst Inpatients in East Suffolk

**DOI:** 10.1192/bjo.2024.529

**Published:** 2024-08-01

**Authors:** Shamim Ruhi, Anne Alase, Motolani Aregbesola, Quratulain Khan, Fariha Rizvi

**Affiliations:** 1 Norfolk and Suffolk NHS Foundation Trust, Ipswich, United Kingdom; 2 Leeds and York Partnership NHS Foundation Trust, Ipswich, United Kingdom

## Abstract

**Aims:**

The use of psychotropics and polypharmacy among patients with learning disability have been widely discussed. Mental illness increases morbidity and mortality and the addition of polypharmacy potentiates these risks.

It is important to determine the proportion of inpatients with psychotropic polypharmacy, highlight associated socio-demographic and clinical factors, and follow up plans for such patients at the point of discharge.

**Methods:**

A retrospective collection of data was completed using electronic records of patients 18 years and above who were discharged from inpatient psychiatric wards located in East Suffolk between 1st July and 31st December 2021.

Data available in discharge medication letters, discharge summaries and inpatient clinical notes were also used in the study.

**Results:**

Amongst 256 inpatient episodes included within the audit, polypharmacy was found in 52% cases.

Of which 80% of patients were above 65 yrs and 56.3% of them were male.

Out of the included episodes, 74% were on combination and 26% were on augmentation therapy.

About 40% had a single diagnosis of schizophrenia/schizophrenia-like delusional disorders, while around 25% had a mood disorder.

9% of episodes had a singular diagnosis of personality disorder and 8.4% of episodes had >1 psychiatric diagnosis.

**Conclusion:**

Despite the increased side effect burden and risks in the presence of physical health co-morbidities, polypharmacy remained prevalent in this group of inpatients.

More than a quarter of patients were on sedative augmentation without any clear plan or recommendation for deprescribing after discharge.

In order to improve clinical practice, more frequent medication reviews should be recommended when there is high prevalence of psychotropic polypharmacy.

